# Benchmarks for ethically credible partnerships between industry and academic health centers: beyond disclosure of financial conflicts of interest

**DOI:** 10.1186/s40169-015-0077-y

**Published:** 2015-12-14

**Authors:** Eric M. Meslin, Joshua B. Rager, Peter H. Schwartz, Kimberly A. Quaid, Margaret M. Gaffney, Jon Duke, William M. Tierney

**Affiliations:** Indiana University Center for Bioethics, 410 West 10th Street, Indianapolis, IN 46202 USA; Regenstrief Institute Inc., 410 West 10th Street, Indianapolis, IN 46202 USA

**Keywords:** Academic-industry partnerships, Collaboration, Ethics, Benchmarks, Cooperative behavior, Academic-industry relationships, Conflict of interest, Academic health centers, Industry

## Abstract

Relationships between industry and university-based researchers have been commonplace for decades and have received notable attention concerning the conflicts of interest these relationships may harbor. While new efforts are being made to update conflict of interest policies and make industry relationships with academia more transparent, the development of broader institutional partnerships between industry and academic health centers challenges the efficacy of current policy to effectively manage these innovative partnerships. In this paper, we argue that existing strategies to reduce conflicts of interest are not sufficient to address the emerging models of industry-academic partnerships because they focus too narrowly on financial matters and are not comprehensive enough to mitigate all ethical risk. Moreover, conflict-of-interest strategies are not designed to promote best practices nor the scientific and social benefits of academic-industry collaboration. We propose a framework of principles and benchmarks for “ethically credible partnerships” between industry and academic health centers and describe how this framework may provide a practical and comprehensive approach for designing and evaluating such partnerships.

## Background

Industry has provided support for research by university-based investigators for decades [1, 2], and from the earliest days of industry-academic collaboration, federal regulators and professional associations have sought to address the potential for conflict of interest by developing policies that would minimize the risk of bias due to decreased scientific objectivity that has tainted some academic faculty who partnered with companies [3]. The level of scrutiny for clinicians and investigators has never been greater than it is now, as evidenced by: tougher rules for accrediting continuing medical education programs [4]; specific provisions in the Affordable Care Act that require corporate sponsors and hospitals to report the funding physicians receive from industry research grants [5]; disclosure requirements by journals [6]; guidelines developed by professional associations [7]; and more structured financial disclosure systems such as the Association of American Medical Colleges’ “Convey” program [8]. Like many Academic Health Centers (AHCs) in the US, our university has engaged in robust discussions on conflict of interest issues, the most recent of which was a thorough revision to Indiana University School of Medicine’s “Industry Relations Policy” [9]. Like most AHC policies, Indiana University’s was developed in light of the current emphasis on disclosing potential conflicts, restricting participation in speaker’s bureaus, limiting access to industry representatives, and setting expectations regarding compensation for meals, travel, and publication and related matters [10, 11].

Yet, as worrisome as financial conflicts of interest by individual researchers may be, and as valuable as such guidance will most certainly be, these restrictions need to be framed against a countervailing force that is becoming more common within the AHC environment: the *active development and encouragement* of AHC-industry research partnerships (AHCIP) and the innovative structures these partnerships might take. Medical schools are increasingly encouraging their research faculty to pursue entrepreneurial strategies, to start companies and to partner actively with industry [11–13].

The rationale for this emphasis is clear. By leveraging the more than $68 billion of research funding by industry [14], these efforts may accelerate research and lower the barriers to developing marketable medicines and other technologies which is ostensibly the central mission of translational science [15]. Indeed, the country’s premier public biomedical research sponsor, the National Institutes of Health, ventured further into this territory [16] in 2014 when it launched its Accelerating Medicines Partnership (AMP) involving 10 major biopharmaceutical companies in its initiative “to transform the current model for developing new diagnostics and treatments by jointly identifying and validating promising biological targets for therapeutics…[and] to increase the number of new diagnostics and therapies for patients and reduce the time and cost of developing them” [17].

More recently, a high profile and somewhat contentious discussion has played out in major medical journals about whether current conflict of interest policies create an unnecessary and undesired chill of collaboration [18, 19]. This discussion has highlighted the persistent tension between the imperative to danger that conflicts of interest will undermine and taint medical research and the imperative to harness all available tools to translate research into ways that combat disease and help patients. Our project aimed to balance these two key issues by establishing a clear process and set of guidelines to evaluate steps taken to control conflicts of interest while also allowing robust and hopefully productive collaboration.

## Common and emerging AHCIP models

The push towards more collaborative research between industry and academic researchers has generated innovative approaches to structuring and managing the formal relationships between the respective organizations. Historically, the typical arrangement involved a private firm, such as a pharmaceutical company, providing a grant or contract to an academic investigator to conduct a targeted study or pursue a line of research that is of interest to the company and also falls within the scientific interests and expertise of the investigator. Indeed, a recent study confirmed that this popular structure continues to persist: the most common type of industry grant supports conventional clinical trials of new drugs or indications, and the most common arrangement is the use of unrestricted grants and fee-for-service structures to clinician-scientists in AHCs [20]. Many examples of these arrangements exist [1, 21].

However, newer types of AHCIPs are emerging that diverge from this model and use collaborative arrangements to *jointly* pursue broad areas of research, data analysis or drug development [12, 21]. Such arrangements may include: corporate venture capital funds, academic drug discovery centers, university consortia, competitive grant processes, and risk sharing [20, 22]. As just one example, in 2012, The Regenstrief Institute, Inc. (RI)–a health services research organization in Indianapolis affiliated with the IU School of Medicine–entered into a 5-year partnership with a large multinational pharmaceutical company the goal of which was to improve “the health of patients through data analytics, health care innovation, education and research that supports evidence-based health care” [22]. Rather than sponsoring one or more projects or researchers, this partnership established a process where a steering committee comprised of both RI and industry representatives sets annual research priorities and then solicits, reviews, and selects diverse research proposals to be funded according to pre-agreed criteria [22]—a process that has certain similarities to the proposal submission and review selection procedures used for government research grants. Essential to submitting a proposal and being funded is that the project must be co-led by a university investigator and an industry scientist. Arrangements like these differ in substantive ways from the more traditional grant-supported-investigator model in terms of study duration, financial structure, expectations of reciprocal benefit, publishing results, and potential for broad impact at both organizations.

## A need for a new tool: evaluating the ethics of AHCIPs

The structural differences between the traditional model and these emerging collaborative AHCIPs suggest that while financial disclosures may be a necessary starting point to identify, assess, and ameliorate potential conflicts of interest and related ethical issues, they are no longer sufficient to address the issues unique to partnerships. However, while the emerging AHCIP model may place a gap between industry money and individual investigators, possibly rendering conflict of interest disclosure policies less useful, this does not address the perception that the involvement of industry with academic *institutions* may pose more pernicious threats to values that AHCs represent and the trust that the public places in them [3, 19].

The emerging AHCIP model would then appear to challenge both the ethical rationale for current conflict of interest policies and the efficacy of current ethical tools for evaluating the relationships themselves. For example, in the traditional model, the principal ethics emphasis is to adequately manage and reduce both the risk of investigator bias in the conduct and reporting of research and the undue influence exerted by the prospect of profiting from industry support [23]. Most efforts that seek to minimize this risk aim to eliminate the conflict by prohibiting investigators from accepting industry funds or by requiring disclosure and management plans [3].

In the emerging AHCIP model however, the ethics emphasis is not solely limited to reducing the risk of bias, but also includes enhancing the positive benefits of the partnerships while mitigating other ethical risks. This aspect of AHCIPs can be best accomplished by anticipating potential ethical issues early in the construction of the partnership.

While much progress is being made to fine-tune guidance for AHC-based investigators to better manage the risk of conflict of interest, comparably less progress has been made in developing an ethics-based approach to anticipating and addressing the suite of issues that arise in the emerging models of AHCIPs. In March 2013, we were asked to review an AHCIP and design such a framework. The Regenstrief Institute (RI), Inc., contracted with the Indiana University Center for Bioethics (IUCB) to assess the RI component of their AHCIP and provide substantive, actionable recommendations to minimize the risk of potential or actual conflicts of interest and address other ethical issues. A key outcome was that the proposed work would be widely shared to allow discussion and critique.

## An ethics assessment of an innovative AHCIP

We began with two presumptions. First, we were open-minded about the potential for AHCIPs to satisfy a set of accepted ethical standards. This alone was controversial, as there is considerable opposition within the bioethics and science communities about this idea. Some consider the very notion of industry collaboration with a university to be ethically untenable at best, and ethically forbidden at worst [23–25]. We reserved judgment. Second, we knew from the outset that no ethics assessment could occur without a framework that was both philosophically sound and practically implementable. The Holy Grail in this case would be an approach to AHCIPs that maximizes the reciprocal science and research goals of both organizations while meeting (or exceeding) the highest levels of external ethical scrutiny. Arrangements that satisfy or demonstratively seek to satisfy these two features we refer to as “ethically credible partnerships.”

If they are to be useful to broader audiences, ethical frameworks to guide AHCIPs must go beyond basic abstract principles. Ethics principles are often used in health and science as general action guides for generating ethical policies [26]. Nonetheless, stand-alone principles do not provide guidance for specific situations without some degree of interpretation, and disagreements often arise at this stage of application. Approaches that are intended to be more systematic have been developed include: checklists, practice guidelines [27], points to consider [28, 29], and benchmarks [30, 31]—all of which are more granular and hence less abstract and more applicable to individual cases than principles alone and thus may serve as achievable metrics.

Benchmarking for ethics content is a recent addition to the suite of planning and evaluation tools in health and science. Norman Daniels employed benchmarks in health care reform that have been widely discussed and used for policy guidance [32, 33]. Similarly, Emanuel et al. developed an ethics framework that combines principles and benchmarks to assess the ethical acceptability of clinical trials in developing countries [30], an approach also embraced by others [34].

We foresaw a unique value to creating ethical benchmarks for AHCIPs. They would (1) establish a practical and achievable floor for academic-industry partnerships; (2) provide more direct guidance than principles alone; (3) allow considerable freedom to accommodate the various ways an individual benchmark may be achieved; (4) enhance AHCIP ethics reviews that go beyond financial conflicts of interest; and (5) enable a holistic approach to prospectively assess progress and identify possible deficiencies so processes and outcomes can be improved. We also intended for this to be an early test of a proof-of-principle for an alternative approach to evaluating AHCIPs beyond conflicts of interest.

## Creating the principles and benchmarks

Our process for creating the principles and benchmarks of ethically-credible AHCIPs involved the reviewing key partnership documents; reviewing the relevant academic literature; scoping the issues; and developing and vetting the draft Principles and Benchmarks before producing a final document. Some commentary on the nature of the Regenstrief AHCIP has been recounted elsewhere which gives an account for how the Principles and Benchmarks were applied [22, 35]. Here we offer a slightly fuller account of these steps.

1. *Key document review* After completing a Confidentiality Agreement, we reviewed key documents including the Master Collaboration Agreement, an internally-produced investigator’s guide to the partnership, and an internal report of a survey of investigator and staff experiences.
2. *Literature review* We conducted several literature reviews on ethical issues in academic-industry collaborations in September 2013. These papers were identified from the published literature using the OVID Medline Medical Subject Headings (MeSH terms): Industry AND Academies and Institutes/or Universities AND Cooperative Behavior AND Ethics, and from Google Scholar and PubMed using similar keywords. No filters regarding country of origin were used. Collectively, these searches resulted in 63 papers which were retrieved, read, and analyzed for eligibility. Papers were excluded if they did not address or report on ethical issues, best practices, challenges, or exemplary models of academic-industry partnerships. This process resulted in 29 papers.
3. *Scoping issues* The team met weekly from October 2013 to December 2013. Each investigator had read the partnership documentation and was assigned a designated set of the retrieved papers to better determine their usefulness for creating the benchmarks, looking specifically for exemplars, best practices, challenges, and suggestions for improving academic-industry partnerships. Discussion of all papers occurred with the entire team to reduce the risks of people with different backgrounds extracting different meaning from the same text [36]. All papers were annotated and compiled into a bibliography available online [37].
4. *Developing and vetting the principles and benchmarks* Following the scoping exercise, all items were transformed into an initial set of 60 benchmarks which we then separated into categories based on relatedness amongst their content. These categories were named and became an initial set of principles which we continually refined through an iterative process.

These principles provided the moral foundation for our benchmarking framework. All appear in various forms in the literature. The team agreed that the principles should be distinct and action guiding, and less abstract than other well-known bioethics principles such as respect for persons, beneficence, nonmaleficence and justice [26]. In their totality we intended for the principles to be sufficient to help AHCs answer the question: what are the actions or policies that ethically-credible AHCIPs ought to take? The final list included 9 principles of ethical credibility:

- *Academic freedom* This principle substantiates the ability of researchers to pursue their independent interests as they see fit [38, 39]. This principle may be satisfied if the partnership both promotes investigator-initiated science regardless of funding source, and protects investigators’ from being coerced into conducting research they are not comfortable pursuing. Additionally, the partnership ought to permit investigators to initiate or continue collaboration with any other qualified group, person, or entity and ensure that investigators involved in the partnership are given equal opportunity to submit proposals for funding.
- *Conflict of interest policy and management* This principle is meant to justify actions which seek to reasonably control the competing or potentially competing interests and commitments of involved parties and persons [40–45]. Managing such conflicts can be achieved through establishing tested methods for assessing potential conflicts of interests. A partnership should also protect students, post-doctoral fellows, and junior faculty involved in partnership projects from exploitation.
- *Data sharing and access* This principle highlights the growing importance and use of ‘big data’, health informatics and database research. [2, 46–48] As such AHCIPs should create mutually agreed upon procedures for accessing each partner’s data and other relevant clinical information in order to facilitate research.
- *Intellectual property* This principle addresses the need to protect the property rights of each partner and investigator that predate the partnership or arise from work conducted within it [2, 46–48].
- *Effective governance* This principle addresses legal issues, administrative duties/obligations, and priority setting and fosters fairness, cooperation, and communication within the partnership [38, 46, 48–50]. Effective governance should be structured in a way that enables research and is minimally burdensome. An ethically credible partnership that exhibits the principle of Effective Governance would establish parameters for what type of projects will and will not be funded; create ways to protect each party from a unexpected end to the partnership; formally assess the efficiency, effectiveness, and achievements of the partnership on an annual basis; and ensure that clear, comprehensive and efficient procedures exist for all governance entities of the partnership and that these are known to all investigators.
- *Protection of human subjects* This principle has been the central justification for the system of oversight of research in the US and in other countries. Its most salient method of ensuring protection has been the use of ethics review bodies, known in the US as IRBs. [51]. This principle binds partners to a commitment to jointly comply with relevant domestic and international requirements for biomedical, behavioral, epidemiological, and health services research as applicable. An ethically credible partnership strives to ensure that all investigators, staff, and other contributing members of the partnership have adequate training in the responsible conduct of research will aid in fulfilling this principle.
- *Publication* This principle arises from a concern regarding how research findings from academic-industry partnerships are published and otherwise disseminated through the peer-reviewed literature and scientific and professional conferences [2, 38, 39, 49, 52, 53]. Establishing publication committees and/or guidelines at the beginning of each AHCIP will avoid most publication and authorship conflicts. An ethically credible partnership should formally establish the right for researchers to publish their results and should also encourage timely dissemination of its research findings.
- *Social, scientific, and industrial value* It is accepted that partnerships should benefit the parties involved by satisfying the particular goals of each partner [2, 3, 38, 54]. It is also accepted that benefits of AHCIPs should extend to science and society more generally. An ethically credible partnership should seek to create value that, overall, will benefit others. Naturally, there are many types of benefits, but there should be agreement that the partnership is better positioned to advance these benefits than the individual partners alone. An ethically credible partnership will also be cognizant of the competitive nature of industry and academia and will seek to reasonably maintain any competitive advantage or expertise a partner might have in a given area of research.
- *Transparency* This principle states that at a minimum, the collaboration’s function and initial agreement are known by and visible to relevant parties and the public [38]. Transparency is primarily achieved through establishing approaches to communicating internally and externally the commitment of the partners to these principles and the procedures implemented to fulfill them. Accordingly, partnerships with other companies and/or academic institutions should be disclosed to each partner.

In contrast with the principles, benchmarks are more concrete and intended to be framed as actionable steps that an organization could use to determine whether or not the principle was being satisfied. The combined document of principles and benchmarks constituted the ethics framework for our review. Benchmarks follow from or are developed in response to the more general principles. We did not establish a required number of benchmarks for each principle *a priori* but sought simply to provide as much guidance as we thought necessary to satisfy a given principle. Accordingly, some principles required more benchmarks than others to better specify what a particular principle necessitated. The resulting principles and benchmarks may be seen as the outcome of a process which sought to be exhaustive and comprehensive in its criteria for ethically credible AHCIPs.

We shared the ethics principles and benchmarks with RI leadership in a focus group setting before finalizing a document consisting of 9 principles and 23 benchmarks. (Table 1).

**Table 1:** Ethical principles and benchmarks for ethically credible partnerships between ahcs and industry

Principles	Benchmarks
Academic freedom	1. Promote investigator-initiated science and protect the ability to attract and maintain federal research support
	2. Permit investigators to initiate or continue collaboration with any other qualified group, person, or entity
	3. Ensure that all investigators involved in the partnership are given equal opportunity to submit proposals for funding
	4. Avoid obligating faculty to work outside their own self-defined scientific area
Conflict of interest policy and management	5. Protect students, post-doctoral fellows and junior faculty involved in collaborative projects from exploitation 6. Ensure that effective mechanisms exist to eliminate, control or manage conflicts of interest in the partnership
Intellectual property	7. Ensure all investigators and both partners retain their proprietary and intellectual property rights throughout and after the partnership
Data sharing, access	8. Ensure that data sharing arrangements are explicit and that all rights to access data are fairly negotiated at the outset of the partnership
Effective governance	9. Establish parameters for what type of projects will and will not be funded (e.g. add-on projects, training, pilot studies)
	10. Create ways to protect each party from an unexpected end to the partnership
	11. Assess formally the efficiency, effectiveness, and achievements of the partnership on an annual basis
	12. Ensure that clear, comprehensive, and efficient procedures exist for all governance entities of the partnership and are known to all investigators
Protection of human subjects	13. Ensure that all investigators, staff and other participants in the partnership have adequate training in the responsible conduct of research and related ethical issues 14. Ensure that all projects in the partnership aim to satisfy the highest ethical standards
Publication	15. Ensure the right of all researchers associated with the partnership to publish
	16. Disseminate all research results at the conclusion of collaborative studies in a timely fashion
	17. Ensure authorship follows ICMJE guidelines
Social, scientific, and industrial value	18. Maintain competitive advantage in the specified research domains 19. Structure the research to maximize potential benefit for communities and society
	20. Structure the partnership to have the best chance of benefiting both partners and harming neither
Transparency	21. Widely publicize the partnership agreement and collaborative opportunities to the public and employees
	22. Establish procedures for frequent and effective communication between partners
	23. Ensure both partners are aware of other partnerships each may be involved in

## The benefits of using principles and benchmarks for building and assessing ethically credible partnerships

In their well-received paper that employed principles and benchmarks for ethically evaluating research in developing countries, Emanuel et al. described four potential benefits of identifying and using ethical benchmarks as tools for ethics evaluation: (1) benchmarks would allow for enhanced clarity in assessing which principle more stringently applies in a particular context; (2) the benchmarks would characterize an explicit and systematic delineation of steps already being taken by conscientious researchers; (3) benchmarks would provide “more-specific and more-practical guidance… that can serve as a reminder and common reference for all those planning, conducting, and evaluating research”; and (4) benchmarks could both narrow disagreements when principles conflict and make these disagreements less ethically worrisome [30].

We found many of the same benefits of benchmarks in our review of the RI AHCIP, and we believe similar benefits may be experienced by other AHCIPs and AHCs who implement this document. Specifically, by agreeing to and targeting these benchmarks in advance, partners can: (1) establish a practical and achievable floor for mutual expectations that can also flexibly accommodate the diversity of AHCIP structures; (2) provide more direct and specific guidance than principles alone; (3) embed ethical considerations throughout the partnership beyond conflicts of interest; and (4) prospectively assess ethical progress and identify possible deficiencies so processes and outcomes can be improved.

Furthermore, we found that specification of the benchmarks allowed a significant amount of versatility and adaptability for various methodologies of ethics review. For instance, the structure of the benchmarks readily allowed for the generation of survey questions. As just one example, as part of a larger survey of RI scientists, we assessed the achievement of benchmark #2 by asking respondents to indicate whether they agreed or disagreed (using a 5-point Likert scale) with the following statement, which is an analog to benchmark #2: “The… partnership ensures that all investigators involved in the partnership are given equal opportunity to submit proposals for funding.”

Additionally, the principles and benchmarks were readily adaptable to the interview guide we used in a focus group early in the vetting process. While we did not employ other qualitative methods such as key informant interviews, we believe that the benchmarks document could also be used to generate questions and probes necessary for such an interview guide were it to be used by others.

We also found that the benchmark framework allows reviewers to identify areas of excellence and deficiency and then prescribe recommendations to address problems. For instance, while some principles and benchmarks were not fully met by the RI AHCIP, we made specific recommendations that, if followed, could assist RI in meeting or exceeding the benchmarks in the future. [35] One illustrative recommendation was to “Increase transparency by providing more opportunities for investigators to become educated about the partnership, especially in areas where lack of understanding could potentially lead to an erosion of trust” [37]. This recommendation was made based on our finding from a benchmarks-based survey of all RI scientists that awareness of the partnership and its policies and procedures was lower than the acceptable ethical threshold we required. Indeed, Daniels et al. found a similar benefit to using their benchmarks for fairness in health care reform, since their benchmarks assisted in identifying “places where proposed reforms were insufficiently detailed or vague about mechanisms to reveal their effects; [and in revealing] problematic assumptions about how goals of reform would be achieved” [32]. A fuller description of the methodology and recommendations we made are available elsewhere [37].

## Assessing the efficacy of principles and benchmarks for ethically-credible AHCIPs

We have described a proof of concept for assessing AHCIPs. We believe this framework could assist others in reviewing AHCIPs of varying structures and research foci. We also believe that applying this framework to other AHCIPs could assist in assessing the quality and efficacy of this framework so as to improve and refine it. In addition, there may be other ways to ascertain if this framework functions as an appropriate evaluative tool for AHCIPs. We hypothesize that this may be accomplished in at least three ways. First, the framework should be considered a success if parties setting up AHCIPs choose to incorporate it in their governance documents, as this would indicate that the benchmarks are taken seriously enough to be given legal or contractual weight. Secondly, the framework will be a success if others adopt the idea that AHCIPs have the potential to be ethically credible, as long as they meet the benchmarks. This alone would be a significant step given the adversarial disposition often taken against *any* industry involvement with academic institutions and research. Thirdly, the benchmarks would prove their merit if they were adopted by accrediting bodies such the AAHRP, or the Joint Commission, or by large not-for-profit entities that work in this area such as PhRMA or the University Industry Demonstration Partnership. It may take time for any or all three of these developments to occur, and there may well be other ways to validate this tool. We can report, as of this writing however, that the RI now incorporates these benchmarks into all of its partnership contracts.

## Conclusion

The impetus to discover, develop, and implement new therapies and medicines is now utilizing the translational science paradigm, although not without certain challenges [55]. Similarly, the incentive to leverage the complementary strengths of industry and AHCs heightens the prospect of new models of mutually beneficial inter-institutional collaboration. Jointly these developments raise the ethics stakes considerably. No longer is society’s moral concern limited to the lone researcher supported by an industry sponsor and the risks of biased influence associated with such support. Now, entire institutions are under the microscope—and rightly so. For this reason, the methods for managing conflict between academia and industry may no longer be attended to by disclosure requirements and other limitations on individual behavior. It requires a new prescriptive approach that strikes a balance between the purported benefits of collaboration and the broader risks, including reputational risks. Our recent experience suggests that a new approach to rigorous scrutiny of academic-industry partnerships is possible, though it is too early to tell whether it could meet the variety of needs in this area. More experience will be required to determine whether prospective ethical benchmarking supports both the benefits of AHCIPs and the highest standards of ethical integrity. We therefore encourage other AHCs contemplating or engaging in industry partnerships to use and improve our benchmarks, and to further develop ethical approaches to the conduct of industry-funded research collaborations.

## Abbreviations

AHCIP: Academic Health Center-Industry Partnership
IUCB: Indiana University Center for Bioethics
RI: Regenstrief Institute, Inc
