# Erratum to: Benchmarks for ethically credible partnerships between industry and academic health centers: beyond disclosure of financial conflicts of interest

**DOI:** 10.1186/s40169-016-0083-8

**Published:** 2016-01-29

**Authors:** Eric M. Meslin, Joshua B. Rager, Peter H. Schwartz, Kimberly A. Quaid, Margaret M. Gaffney, Jon Duke, William M. Tierney

**Affiliations:** Indiana University Center for Bioethics, 410 West 10th Street, Indianapolis, IN 46202 USA; Regenstrief Institute Inc., 410 West 10th Street, Indianapolis, IN 46202 USA

## Erratum to: Clin Trans Med (2015) 4:36 DOI 10.1186/s40169-015-0077-y

Following publication of the original version of this article in *Clinical and Translational Medicine* [1] it was brought to our attention that the name of one author was spelled incorrectly. The name of author William M. Tierney was incorrectly spelled as William H. Tierney. The original article has been updated with the correct information. We apologize for the inconvenience.
